# Ensemble Machine-Learning-Based Prediction Models for the Compressive Strength of Recycled Powder Mortar

**DOI:** 10.3390/ma16020583

**Published:** 2023-01-06

**Authors:** Zhengyu Fei, Shixue Liang, Yiqing Cai, Yuanxie Shen

**Affiliations:** School of Civil Engineering and Architecture, Zhejiang Sci-Tech University, Hangzhou 310018, China

**Keywords:** recycled powder mortar, compressive strength, machine learning, SHAP

## Abstract

Recycled powder (RP) serves as a potential and prospective substitute for cementitious materials in concrete. The compressive strength of RP mortar is a pivotal factor affecting the mechanical properties of RP concrete. The application of machine learning (ML) approaches in the engineering problems, particularly for predicting the mechanical properties of construction materials, leads to high prediction accuracy and low experimental costs. In this study, 204 groups of RP mortar compression experimental data are collected from the literature to establish a dataset for ML, including 163 groups in the training set and 41 groups in the test set. Four ensemble ML models, namely eXtreme Gradient-Boosting (XGBoost), Random Forest (RF), Light Gradient-Boosting Machine (LightGBM) and Adaptive Boosting (AdaBoost), were selected to predict the compressive strength of RP mortar. The comparative results demonstrate that XGBoost has the highest prediction accuracy when the a10-index, MAE, RMSE and R^2^ of the training set are 0.926, 1.596, 2.155 and 0.950 and the a10-index, MAE, RMSE and R^2^ of the test set are 0.659, 3.182, 4.285 and 0.842, respectively. SHapley Additive exPlanation (SHAP) is adopted to interpret the prediction process of XGBoost and explain the influence of influencing factors on the compressive strength of RP mortar. According to the importance of influencing factors, the order is the mass replacement rate of RP, the size of RP, the kind of RP and the water binder ratio of RP. The compressive strength of RP mortar decreases with the increase in the RP mass replacement rate. The compressive strength of RBP mortar is slightly higher than that of RCP mortar. Machine learning technologies will benefit the construction industry by facilitating the rapid and cost-effective evaluation of RP material properties.

## 1. Introduction

During the rapid process of the urbanization of the world, a large amount of construction and demolition (C&D) waste has been generated; for example, United States and China produce 700 and 1800 million tons of C&D waste each year, respectively [1]. C&D waste is usually disposed of by dumping and landfilling, which not only causes air and soil pollution but also enforces enormous pressure on limited landfills [2]. Since most of the C&D waste can serve as replaceable materials to aggregates and mortar, the recycling of C&D waste is a promising method of conserving natural resources [3].

There have been massive researches on the resourceful reuse of C&D waste, which can be generally divided into two categories: recycled aggregate concrete (RAC) and recycled powder concrete (RPC). At present, there are substantial experimental, numerical and theoretical studies on the strength of RAC. Researchers proposed methods based on aggregate skeleton theory, gray correlation analysis, finite element analysis and so on to predict the compressive strength of RAC and analyze its influencing factors [4,5,6,7].

**Figure 1:**
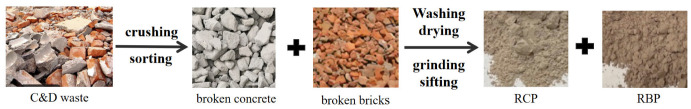
Recycled powder preparation flow chart.

Recycled powder (RP) is a by-product of recycling C&D waste. The process of recovering RP from C&D waste can be divided into three stages: collecting C&D waste, sorting the raw materials and grinding into powder. Figure 1 shows the RP preparation flow chart. The research of RP is still in the initial stage, and there are few empirical formulas for mortar strength. The research on RP is mainly about recycled concrete powder (RCP) and recycled brick powder (RBP) [8]. Researchers have performed various experiments to study the physical and mechanical properties of RCP and RBP in cement to explore the potential of RP as a cementitious material for the preparation of concrete [9,10,11,12,13]. A summary is that, although there are relevant experimental studies, the prediction accuracy and generalization performance on the strength of RPC are still unsatisfactory [14].

Spurred by the constantly challenging requirements of the construction industry, researchers used machine learning (ML) to predict the compressive strength of new types of concrete, such as bio-concrete [15], recycled aggregate concrete [16] and fiber concrete [17]. In addition, ML can be applied to predict the mechanical properties and durability of reinforced concrete structures and to evaluate their service life. At present, some scholars have used ML models to analyze reinforced concrete beams [18], squat reinforced concrete walls [19], reinforced concrete slabs and other engineering problems [20,21]. Whatever the accuracy, however, the black-box nature of the predictions makes ML models unexplainable. The emergence of SHapley Additive exPlanation (SHAP) can solve this problem. SHAP can reasonably explain the interaction of variables in the ML model and the influence of eigenvalues on the results. Researchers have applied explicable ML combined with the SHAP method to concrete to predict the mechanical properties, durability and working performance of reinforced concrete beam [22], slab [23] and column members [24] and to explain the prediction process. The application of ensemble learning in concrete structures is of great interest to researchers [25,26,27,28,29]. Ensemble learning is combining multiple individual learners into one learner to complete a learning task. According to the generation of individual learners, the current ensemble learning methods can be roughly divided into three categories: boosting, bagging and stacking. Ensemble learning integrates several learning devices to achieve better performance than a single learning device [30,31,32]. The three kinds of ensemble learning are widely used in concrete structures. Vimal [33] et al. predicted the compressive strength of concrete with the boosting ML algorithm. Ahmad [34] et al. used the bagging model to predict the compressive strength of concrete containing supplementary cementing materials. Gupta [35] et al. estimated the compressive strength of geopolymer composites by using the stacking model.

All studies on the compressive strength of RP mortar have been conducted through experiments. To the author’s knowledge, no studies have been carried out (1) using an integrated ML model to predict the compressive strength of RP mortar, (2) explaining the prediction mechanism of compressive strength based on ML, or (3) on the factors affecting the compressive strength of the RP mortar. This paper aims to: (1) develop an integrated ML algorithm model to predict the compressive strength of RP mortar and to (2) explain the contribution of input variables to the prediction results. Therefore, four different ensemble ML-based algorithms are developed to predict the compressive strength of the RP mortar and the underlining relationship between input influential factors and output strength is also revealed. This research consists of four stages: firstly, 204 groups of RCP and RBP mortar compressive strength test results are collected from the relevant literature; secondly, eXtreme Gradient-Boosting (XGBoost), Random Forest (RF), Light Gradient-Boosting Machine (LightGBM) and Adaptive Boosting (AdaBoost) algorithms are applied, and then the hyperparameters are optimized to establish the strength prediction model; thirdly, the accuracy and generalization ability of the ML-based models are evaluated with the a10-index, root mean square error, mean absolute error and determination coefficient; finally, SHAP is used to illustrate the prediction process of the best algorithmic model and to investigate the interpretability of the influencing factors. The RP mortar compressive strength test requires a lot of manpower, material resources and time costs. The ML method only needs data to predict the compressive strength and does not need experimental research to test the comprehensive impact of RP mortar compressive strength and to provide reference value for potential users of RP.

## 2. Data Collection and Analysis

Lots of RP mortar test data are required to establish the compressive strength prediction model. To this end, 125 groups of RCP mortar strength and 79 groups of RBP mortar strength data are collected from previous literature. The cement used in the datasets is P.O 42.5 or P.O 42.5R. The complete dataset and its resource are listed in Appendix A.

**Figure 2:**
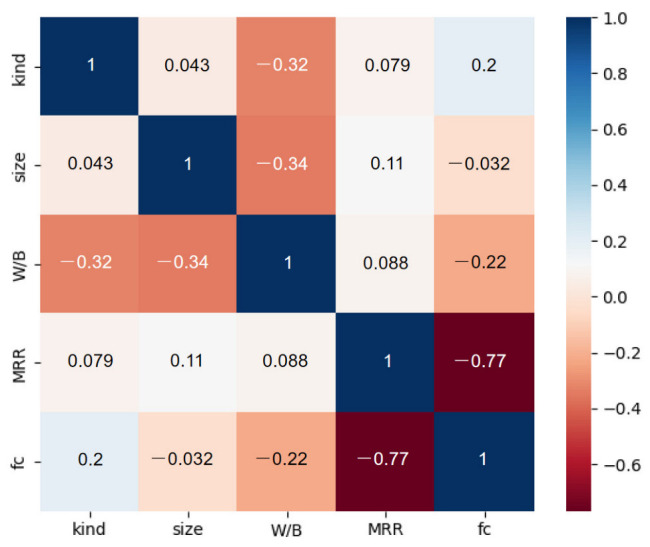
Person correlation coefficient plot of the variable.

**Figure 3:**
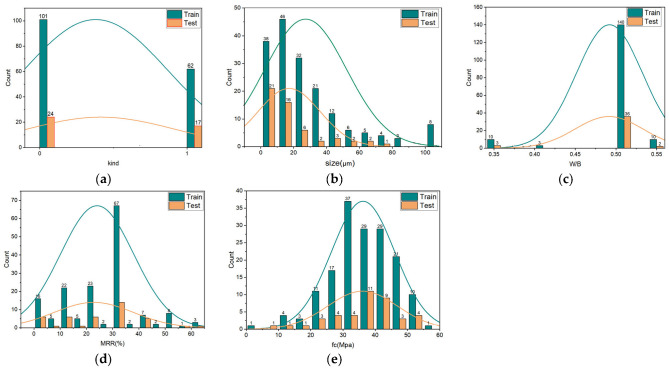
Cumulative plot of the distribution of variables. (**a**) kind; (**b**) size; (**c**) W/B; (**d**) mass replacement rate; (**e**) mortar compressive strength.

It should be noted that the acceptable numbers of data for ML modeling should be greater than 10 times the number of input variables [36]. According to the literature review, four variables are selected as input variables: mass replacement rate (MRR) of RP (%), kinds of RP, water-to-binder ratio (W/B), particle size of RP (μm) and mortar compressive strength (*f*_c_) of RP(Mpa) served as output variables [37,38]. Therefore, the data size (204) chosen in this paper meets the requirement of data number for ML modeling. Figure 2 depicts the Pearson correlation coefficient plot for each variable. It is found that the compressive strength of RP mortar is strongly correlated with MRR and negatively correlated. However, the correlation degree of other variables is considered a weak correlation or no correlation. In addition, the distribution of all variables is shown in Figure 3, and the cumulative percentage of each variable is shown in the orange curve in Figure 3. The distribution of each variable is described below; 125 groups of RCP, accounting for 61%, are classified as kind 0; 79 groups of RBP, accounting for 39%, are classified as kind 1. The particle size of RP refers to the average particle size of RP; the distribution range is 0–100 μm, and the main distribution is in the range of 10–30 μm. The W/B is 0.35, 0.4, 0.5 and 0.55, with 0.5 accounting for the highest proportion [39,40]. The mass replacement rate of RP ranges from 0 to 60%, with 30% being the most frequently used in the experiment [41]. As the output variable, the mortar compressive strength ranges from 16.60 to 56.80 Mpa, and most of them are distributed between 30 and 50 Mpa.

## 3. Machine-Learning-Based Models for Compressive Strength

### 3.1. Data Preprocessing

After collecting the raw data, a detailed dataset can be obtained after filling in the missing values and processing the outliers [42]. However, various metrics have different properties (continuous, discrete) and orders of magnitude, thus training directly will weaken the impact of data with lower orders of magnitude, so the data also need to be normalized (target values usually do not need to be scaled) to allow the data to be trained. Data standardization is the process of turning dimensioned data into dimensionless data and processing data of different magnitudes to the same magnitude, so that data of different dimensions can be compared, which can improve the prediction effect of the model for ML. The formula for z-score normalization can be expressed as:

(1) $$x^{\prime }=\left(x-\mu \right)/\sigma$$

where x is the raw datum, μ is the mean of the data, and σ is the standard deviation [43].

### 3.2. ML Model Algorithm

The model was developed using Python code in the PyCharm (version 2017.1) software, which comes with a set of tools to help users improve their productivity when developing in the Python language, version 3.6.4 of which was used.

**Figure 4:**
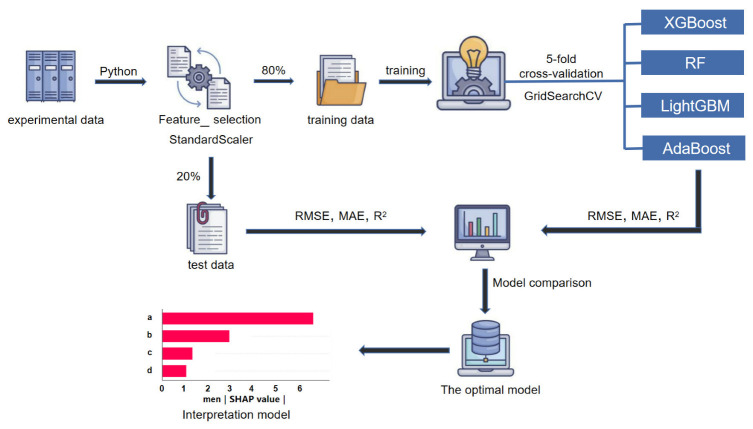
ML algorithm flowchart.

Four ensemble ML algorithms XGBoost, RF, LightGBM and AdaBoost are selected among a handful of ML algorithms to predict the compressive strength of the RP mortar. Ensemble learning algorithms accomplish learning tasks by building and combining multiple machine learners, so they can often obtain superior generalization performance than a single learner [44]. At present, there are three groups of ensemble learning algorithms: bagging-based algorithms, boosting-based algorithms and stacking-based algorithms; RF [45,46] is a typical kind of bagging-based representative algorithms, while boosting-based representative algorithms include AdaBoost, XGBoost, LightGBM, etc. [47]. The stacking algorithm refers to the combination of multiple basic learning tools to generate final predictions that are more accurate than a single stacking model. For example, the SVR, XGBoost and GBDT algorithms can be used to build a stacking algorithm [48]. In this section, the four ML algorithms used in the experiment are briefly explained, which helps to understand the principles and compare their differences. Figure 4 depicts a flowchart of the research method in this paper.

#### 3.2.1. eXtreme Gradient-Boosting (XGBoost)

**Figure 5:**
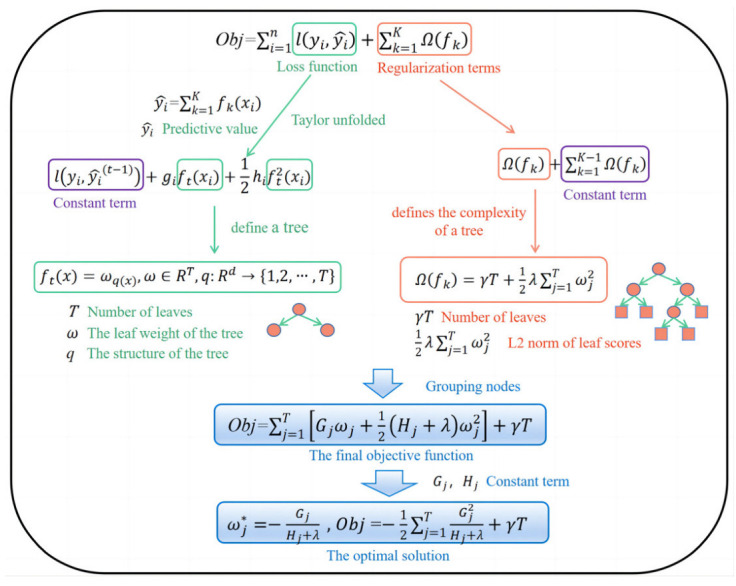
XGBoost algorithm derivation flowchart.

XGBoost is an efficient, flexible and light gradient-boosting decision tree algorithm [49]. XGBoost has the same basic idea as GBDT, but XGBoost has a lot of optimizations and is different from light gradient-boosting [50]. For example, the second-order Taylor formula expansion is used to optimize the loss function and improve the calculation accuracy. A regularization term is used to simplify the model to avoid overfitting. The blocks storage structure is adopted, and parallel computing is implemented. As a forward addition model, it is critical to adopt the ensemble idea (boosting), which integrates multiple weak learners into a strong learner through certain methods. Figure 5 demonstrates the derivation steps of the XGBoost algorithm.

#### 3.2.2. Random Forest (RF)

**Figure 6:**
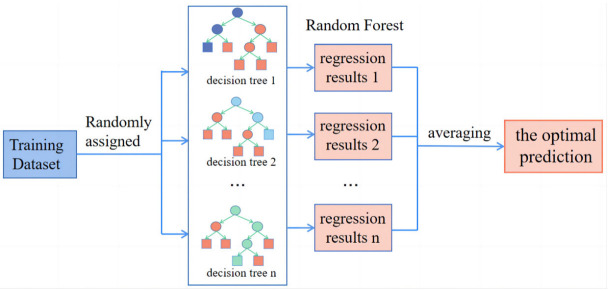
RF modeling flowchart.

RF adopts the idea of bagging [51] with the following steps: (1) each time a sample is taken back for training to form a new training set; (2) adopt the new training set to train *n* sub-models; (3) for regression problems, the predicted value is obtained by using a simple averaging method [52]. The training modeling process of the RF method is shown in Figure 6. RF takes decision trees as the basic unit, and a lot of decision trees are integrated to form a random forest [53,54].

#### 3.2.3. Light Gradient-Boosting Machine (LightGBM)

**Figure 7:**
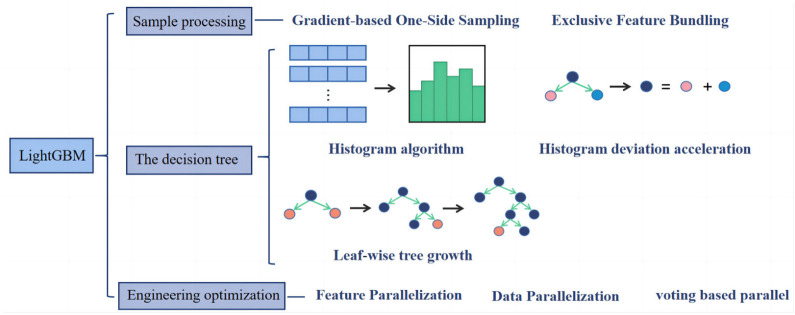
Schematic diagram of LightGBM optimization methods.

LightGBM is also an efficient implementation of GBDT. In principle, lightGBM is similar to XGBoost, but XGBoost consumes considerable space and time. In order to avoid the defects of XGBoost, lightGBM is optimized on the traditional GBDT algorithm [55,56]. LightGBM is a decision tree algorithm based on a histogram, which can reduce memory footprint and computation times. Gradient-based One-Side Sampling (GOSS) is used to exclude most samples with a small gradient, and only the remaining samples are used to calculate the information gain, so as to balance the reduction of data volume and ensure accuracy. Using Exclusive Feature Bundling (EFB), many mutually exclusive features are bound to a single feature to reduce dimensionality. The leaf-wise algorithm with depth restriction is used to control the complexity of the model and to ensure high efficiency while preventing overfitting [57]. Figure 7 shows the schematic diagram of the LightGBM optimization methods.

#### 3.2.4. Adaptive Boosting (AdaBoost)

AdaBoost is also called reinforcement learning or the promotion method, which is proposed by Freund et al. [58]. Its self-adaptation is reflected in each training data set; each sample prediction is assigned a weight, and the wrong prediction is identified and further assigned to the next basic learner with a high weight for this wrong prediction, while the weight of the correctly predicted sample will be reduced. Meanwhile, in each iteration, a new weak learner is added, and the final strong learner is not determined until a predetermined error rate is small enough or until the predetermined maximum number of iterations is reached. However, AdaBoost is sensitive to abnormal samples, and abnormal samples may get higher weights in iteration, which may affect the prediction accuracy. The expression for AdaBoost is Equation (2) below.

(2) $$f\left(x\right)=\overset{N}{\underset{i=1}{\sum }}\left(ln\frac{1}{\alpha_{m}}\right)g\left(x\right)$$

where $g\left(x\right)$ is the median of all weak learners $\;\alpha_{m}G_{m}\left(x\right),\;m=1,2,\ldots ,N.$

### 3.3. Model Tuning and Evaluation

#### 3.3.1. Model Tuning: K-Fold Cross-Validation

**Figure 8:**
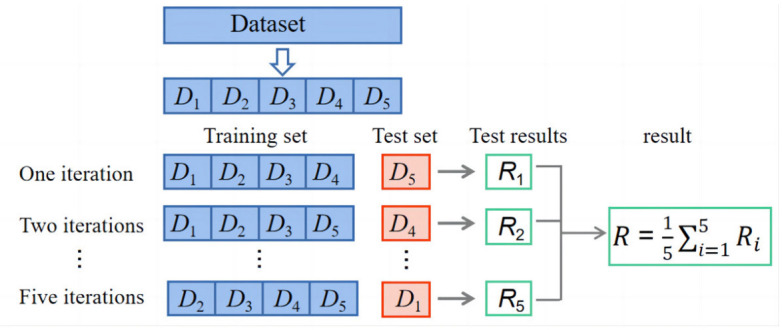
Five-fold cross-validation diagram.

When conducting ML research, the raw data are divided into training and test sets, and the algorithm is evaluated by simple cross-validation. However, the data are only used once; it is not fully utilized, and the division ratio of the raw data has a large impact on the evaluation index of the test set. Therefore, k-fold cross-validation is required, which can solve the problem of not having enough data in the dataset and can also solve the problem of parameter tuning [59]. In this study, five-fold cross-validation is used. Firstly, all samples are divided into five subsets with equal number of samples. Then, the five subsets are traversed successively, and the current subset is served as the validation set each time, and all the remaining samples are used as the training set to train and evaluate the model. Finally, the average value of the five evaluation indices is taken as the final evaluation index [60]. The schematic five-fold cross-validation diagram is given in Figure 8.

#### 3.3.2. Model Evaluation

In order to evaluate the predictive performance of the algorithm used in this study, three evaluation metrics are selected, namely, root mean square error (RMSE), mean absolute error (MAE) and coefficient of determination (R^2^) [61]. RMSE is a measure of the deviation between observed values and true values, also known as standard error. Its significance is that, after the root number of the mean square error, the result of the error is at the same level as the data, and the data can be better described. RMSE is sensitive to the reflection of very large or small errors in a set of measurements, so RMSE can well reflect the precision of the measurement. MAE is used to evaluate the degree of proximity between predicted results and true values, and it is the most easily understood regression error indicator. The smaller the value, the better the fitting effect. R^2^ can be understood as the proportion of the variability of the dependent variable that can be explained by the independent variable through the multiple regression equation. It measures the degree of interpretation of each independent variable to the change of the dependent variable. Its value is between 0 and 1. The closer its value is to 1, the better the regression fitting effect of the variable is. On the contrary, the worse the regression fitting effect is. Furthermore, a following new engineering index, the a10-index evaluates artificial intelligence models by showing the number of samples that fit the prediction values with a deviation of ±10% compared to experimental values. The index is used to evaluate the reliability of the ML model [62]. Equations (3)–(6) are the evaluation of three indicators:

(3) $$RMSE=\sqrt{\frac{1}{m}\sum_{i=1}^{m}{\left(f\left(x_{i}\right)-y_{i}\right)}^{2}}$$

(4) $$MAE=\frac{1}{m}\sum_{i=1}^{m}\left|f\left(x_{i}\right)-y_{i}\right|$$

(5) $$R^{2}=1-\frac{\sum_{i=1}^{m}{\left(f\left(x_{i}\right)-y_{i}\right)}^{2}}{\sum_{i=1}^{m}{\left(\bar{y_{i}}-y_{i}\right)}^{2}}$$

(6) $$a10-index=\frac{m10}{m}$$

where $f\left(x_{i}\right)$ is the predicted value, $y_{i}$ is the target value, and $\bar{y_{i}}$ is the average of the target values, i=1, 2, 3, …, m. m is the number of dataset sample, and m10 is the number of samples with a value of the ratio between the experimental and predicted values between 0.90 and 1.1.

## 4. Model Results and Discussion

All the samples are randomly divided into a training set and a test set according to the proportion of 8:2. The training set is Appendix B, and the test set is Appendix C. In order to ensure the comparability of performance evaluation, the same training and test sets are used for all ML algorithms, hyperparameters are tuned by using grid search combined with five-fold cross validation, and the comprehensive performance is evaluated by using four evaluation metrics (RMSE, MAE, R^2^, a10 − index).

### 4.1. Hyperparameter Settings and Optimization

In order to obtain higher prediction accuracy and generalization ability, the parameters and hyperparameters of the ML algorithm model are adjusted. Parameters refer to the numbers of optimized algorithms, such as the least squares method or the gradient descent method. Model parameters are configuration variables within the model that are usually not set manually by the programmer and whose values can be estimated from the data. Hyperparameters are artificially set parameters before machine learning begins, whose values cannot be estimated from the data, and are often used to help estimate model parameters. Common hyperparameter optimization methods include grid search, random search and Bayesian optimization [63,64]. As there are few hyperparameters that need to be tuned in this study, grid search combined with five-fold cross validation is used to optimize the hyperparameters. Grid search refers to pre-defining a set of potential values for the desired hyperparameters. Each hyperparameter is then assigned a value from the set of potential values to form a different combination of hyperparameters. Then, the machine learning model is trained and evaluated for each hyperparameter combination, and the optimal combination is found during the tuning process [65]. Table 1 lists the major hyperparameter potential values used by each ML model in grid search. In order to determine the final prediction model, its prediction performance needs to be tested by five-fold cross validation [20]. Table 2 presents the values of the major hyperparameters of the ML model, as well as the mean scores of five-fold cross-validation.

### 4.2. Model Prediction Results

**Figure 9:**
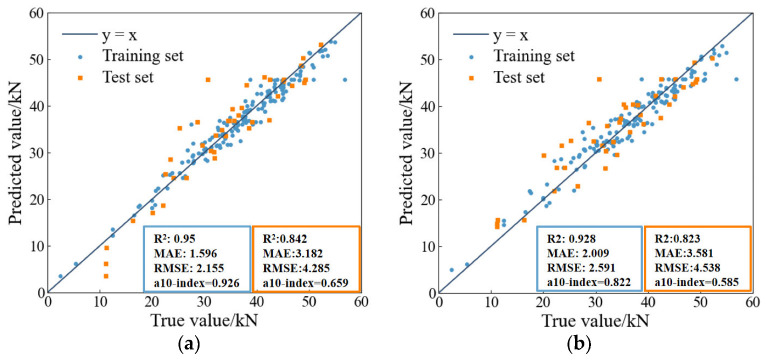

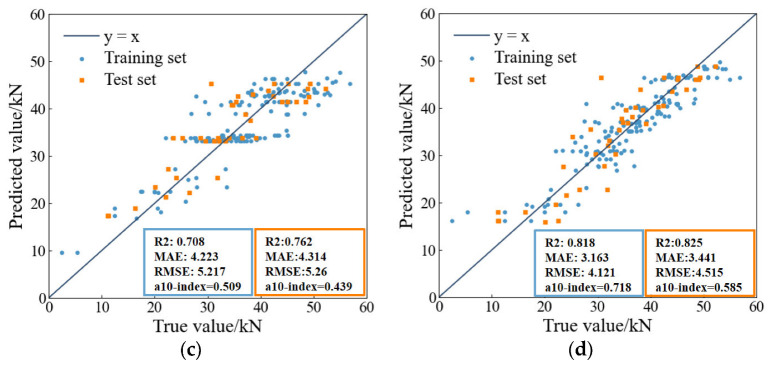
Comparison of the ML models. (**a**) XGBoost. (**b**) RF. (**c**) AdaBoost. (**d**) LightGBM.

The prediction results shown in Figure 9a can be used as a reference for the comparison of generalization ability among the four ML models; blue dots represent the training set results, and orange dots represent the test set results. The data point in the scatterplot of the four ensemble models are distributed around the baseline (y = x). Figure 9a demonstrates the XGBoost-predicted results. It is found in Figure 9a that the difference between the test values and the predicted values is small, and it shows good performance in predicting the compressive strength of the RP mortar. In order to compare the prediction results of the proposed ML models, Table 3 lists the specific evaluation metrics (R^2^, RMSE, MAE, a10-index) of the training and test sets of the four ML models. Using the same training set and test set for prediction, it is found that the R^2^ value of XGBoost is higher than that of the other three ML models, which significantly indicates that XGBoost has the highest prediction accuracy. The evaluation criterion of RMSE and MAE is the smaller the better. It can be seen from the Table 3 that the RMSE and MAE evaluation indices of the XGBoost training set and test set are lower than those of the other three ML models, while the RMSE and MAE of the AdaBoost training set and test set are the largest, indicating that the error of the XGBoost model is the smallest. The evaluation criterion of the a10-index is that the closer it is to 1, the better the reliability will be. It can be seen from Table 3 that the reliability of XGBoost is higher than that of other models and that AdaBoost performs the worst. According to the comprehensive consideration of the four evaluation indices of the training set and the test set prediction model, the accuracy and generalization of the four ML models from high to low are ranked as XGBoost, RF, LightGBM and AdaBoost.

## 5. Interpretability of the Model

Based on the above evaluation results, the XGBoost model is selected for interpretation and analysis. ML algorithm model can only obtain the final prediction results, unable to explain the characteristics and the influence of the predicted results. Therefore, SHAP is applied to interpret each variable on a global and individual scale in the following sections.

### 5.1. Features Explained: Shapley Additive exPlanations (SHAP)

The interpretation methods of the ML model include the linear model, PDP, SHAP, ALE, etc. Among them, SHAP is widely applicable to the interpretable field, which not only reflects the influence of all features of each sample but also shows the positive and negative of the influence [66]. Therefore, the analysis of the research process of the ML regression prediction model in this study is done by SHAP technology. The SHAP interpretation method is to calculate the Shapley value according to the alliance game theory [67]. The eigenvalues of the data instance act as participants in the federation (collection). The Shapley values reveal how much eigenvalues contribute to the prediction [68,69]. The SHAP value follows the following equation.

(7) $$y_{i}=y_{\mathrm{base}}+f\left(x_{i,1}\right)+f\left(x_{i,2}\right)+\ldots +f\left(x_{i,k}\right)$$

where $y_{\mathrm{base}}$ is the baseline of the whole model (usually the mean of the target variables of all samples), $x_{i,k}$ is the k feature of the i sample, $f\left(x_{i,k}\right)$ is the SHAP value of $x_{i,k}$, when $f\left(x_{i,k}\right)$ > 0, indicating that the feature improves the predicted value and has a positive effect. Conversely, it means that the feature makes the predicted value lower and has the opposite effect.

### 5.2. Individual Interpretation

**Figure 10:**
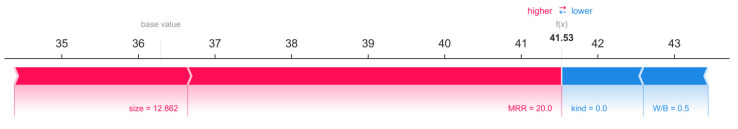
Individual interpretation of SHAP for a sample.

**Figure 11:**
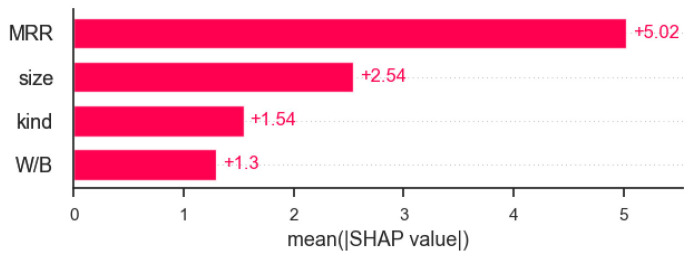
SHAP value bar plot for XGBoost global interpretation.

Figure 10 shows a typical individual prediction graph for a sample. The SHAP value quantifies the local interpretation of the model, using the sum of the effects of each input variable to explain each prediction (Figure 10). The base value represents the mean value of the predicted value; the length of the bar represents the SHAP value of each eigenvalue; red represents the positive influence of the eigenvalue on the predicted value, and blue represents the negative influence. As can be seen from Figure 10, for this sample, starting from the base value, the SHAP values of the four eigenvalues are superimposed to get the result. The mass replacement rate of RP has the greatest positive influence on the prediction of the compressive strength of te RP mortar. Note that it is likely to vary the importance factor for individual predictions (Figure 10) from the mean SHAP value (Figure 11) as the mean value is a global indication.

### 5.3. Global Interpretation

Passing the SHAP values to the bar plot function creates a global feature importance plot, where the global importance of each feature is treated as the average absolute value of that feature across all given samples, as shown in Figure 11. It shows that the mass replacement rate (MRR) of RP has a significant contribution to the prediction of compressive strength of RP mortar, and its SHAP value is twice that of its size, and kind has the least influence on the predicted results. The water–binder ratio in the data set is more concentrated, so it has the least influence on the prediction results.

**Figure 12:**
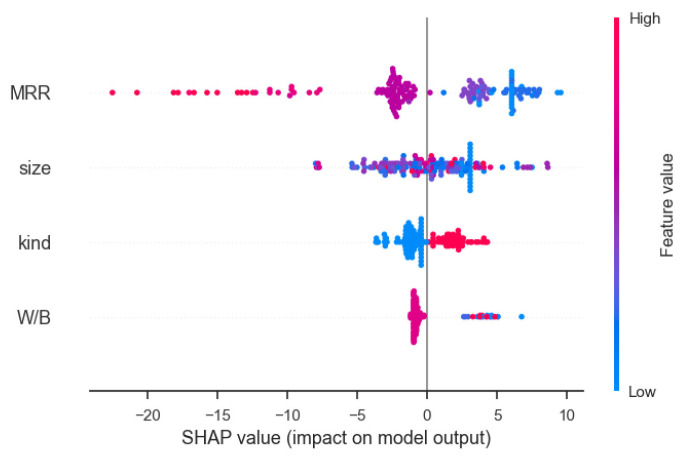
SHAP value summary plot for XGBoost global interpretation.

Each row in Figure 12 represents a feature that is sorted by importance from top to bottom, with the abscissa is displayed the SHAP value. A dot represents a sample, and the change in color from blue to red indicates that the value of the feature itself changes from small to large. Through the overall analysis diagram of features, it can be intuitively seen that the mass replacement rate (MRR) of RP is an important influencing factor, and its feature value is basically negatively correlated with the SHAP value, indicating that the mass replacement rate of RP has a negative impact on the compressive strength of the mortar. The influence of kind on the prediction result is that the eigenvalue is positively correlated with the SHAP value. The characteristic value of RCP is 0 and that of RBP is 1, indicating that the compressive strength of RBP mortar is generally higher than that of the RCP mortar.

### 5.4. Feature Interactions

**Figure 13:**
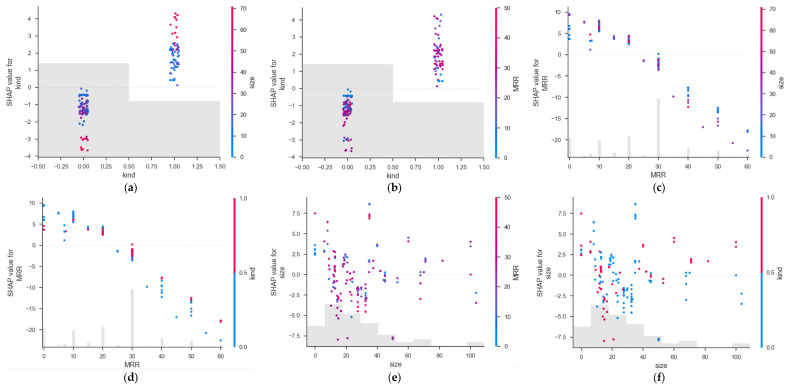
Feature dependency graph. Feature dependence plots for (**a**,**b**) kind; (**c**,**d**) mass replacement rate; (**e**,**f**) size.

Figure 13 depicts the relationship between a given variable and the SHAP value and frequently interacting variables. Each point represents a sample in the feature correlation graph, where the color represents the size of the feature value on the right. The experimental data of the water–binder ratio are concentrated, so the feature interaction analysis is not carried out. The kind, size and mass replacement rate affect each other. It can be seen from Figure 13a that when kind = 0, the SHAP value of kind decreases with the increase in size, while, when kind = 1, the SHAP value of kind increases with the increase in size, indicating that size has an effect on species. It can be seen from Figure 13c,d that, with the increase in the RP quality replacement rate (MRR), the SHAP value decreases to varying degrees. Therefore, as a major factor in reducing compressive strength, the reduction in the SHAP value means the reduction of compressive performance. Some experimental evidence supports these findings [70,71]. It is difficult to obtain obvious results from the figure on the influence of particle size on the compressive strength of mortar, which needs further study in the future.

### 5.5. Sensitivity of Feature

**Figure 14:**
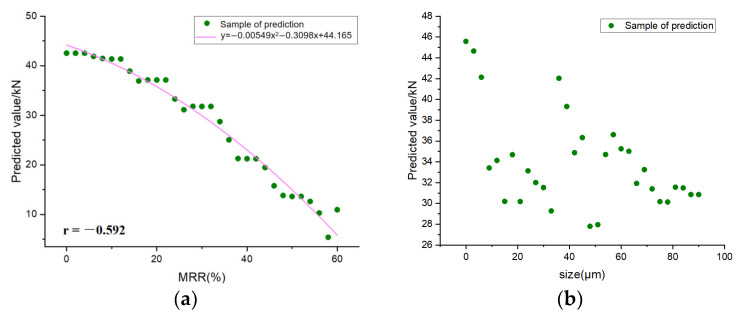
RCP feature sensitivity analysis diagram. (**a**) mass replacement rate; (**b**) size.

**Figure 15:**
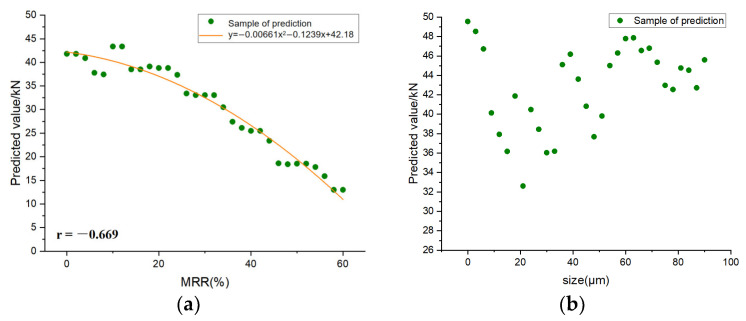
RBP feature sensitivity analysis diagram. (**a**) mass replacement rate; (**b**) size.

Sensitivity analysis is a method used to study how the input changes of ML models affect the output changes [72]. Because the XGBoost model is the best in predicting the compressive strength of RP mortar, XGBoost is selected for sensitivity analysis. In order to investigate the sensitivity of the selected ML model, one feature value is disturbed each time for two kinds of RP, and the average value of the other two feature values remain unchanged so as to predict the compressive strength of the RP mortar. Figure 14 and Figure 15. show the feature sensitivity analysis diagrams of RCP and RBP, respectively. It can be seen from the correlation coefficients −0.592 and −0.669 that the mass replacement rates of RCP and RBP are moderately negatively correlated with the predicted mortar compressive strength. According to the fitting equation, the decline trend of RCP is steeper than that of RBP. There is no obvious linear relationship between the particle size of the two kinds of RP and the predicted compressive strength of the mortar. Due to the concentration of water–binder in the data, sensitivity analysis of the water–binder ratio was not carried out. By comparing the predicted values of the two kinds of RP under different disturbance feature values, it is found that the mass replacement rate of the regenerated powder leads to the biggest change in the predicted results.

(8) $$r=\frac{\sum_{i=1}^{n}\left(X_{i}-\bar{X}\right)\left(Y_{i}-\bar{Y}\right)}{\sqrt{\sum_{i=1}^{n}{\left(X_{i}-\bar{X}\right)}^{2}}\sqrt{\sum_{i=1}^{n}{\left(Y_{i}-\bar{Y}\right)}^{2}}}$$

where *r* is the correlation coefficient, a measure of linear correlation between variables. $X_{i}$ is the feature value of the perturbation, and $Y_{i}$ is the predictive value. $\bar{X}\;$is the average of the feature values of the perturbation, and $\bar{Y}$ is the average of the predicted values.

## 6. Conclusions

In this paper, four ensemble ML models (XGBoost, RF, LightGBM, AdaBoost) are applied to the prediction of the compressive strength of RP mortar. Four variables are used as inputs, including W/B, particle size of RP (μm), kind of RP and mass replacement rate of RP (%). The RMSE, MAE, R^2^ and a10-index are used to compare the performance of four ML models for predicting the compressive strength of the RP mortar. Finally, the SHAP algorithm is used to explain the model prediction and analyze the influence of the input variables on the output. The following conclusions can be drawn from the study:

1. After the comparison of four performance indicators, it is found that XGBoost achieves the best results in four ML models. The a10-index, RMSE, MAE and R^2^ are 0.926, 2.155, 1.596 and 0.95 in the training set and 0.659, 4.285, 3.182 and 0.842 in the test set, respectively, indicating that the XGBoost model is the best model for predicting the compressive strength of the RP mortar.
2. Among the four ML models used in this paper, AdaBoost has the worst performance, with the R^2^ value in the training set only 0.708. This is because AdaBoost is sensitive to abnormal samples, which may gain higher weights in iterations, affecting the prediction accuracy of the final strong learner.
3. SHAP is an additive interpreter that adds up the contribution values of each influencing factor to obtain the final predicted value. In the first place, SHAP provides a global interpretation of the strength prediction and sorts the feature importance of the four input variables to conclude that the mass replacement rate of RP has the greatest influence on the prediction process, which is consistent with the results found in previous experiments [73]. On the contrary, W/B has the least effect on the predicted results, because the W/B used in the experiment is more concentrated.
4. In the feature dependence analysis, the SHAP value decreases with the increase in the mass replacement rate, and the SHAP value of RBP is slightly higher than that of RCP. These findings provide reference value for future research into recycled powder.

## Figures and Tables

**Table 1:** The potential values of major hyperparameters of the machine learning model.

Hyperparameters	Potential Values		
ML Models	Number of Weak Learners	Learning Rate	Maximum Depth
XGBoost	50, 60, 70, …, 200	0.1, 0.2, 0.3, …, 1	1, 2, 3, …, 10
RF	50, 60, 70, …, 200	-	1, 2, 3, …, 20
LightGBM	50, 60, 70, …, 200	0.1, 0.2, 0.3, …, 1	1, 2, 3, …, 20
AdaBoost	50, 60, 70, …, 200	0.1, 0.2, 0.3, …, 1	-

**Table 2:** Values of major hyperparameters of ML models used for resistance prediction.

Hyperparameters	Values			
ML Models	Number of Weak Learners	Learning Rate	Maximum Depth	Average Validation Outcome
XGBoost	100	0.1	6	0.75
RF	100	-	10	0.646
LightGBM	100	0.4	15	0.701
AdaBoost	100	0.3	-	0.628

**Table 3:** Performance of ML models.

Data Set	Training Set				Test Set			
ML Models	XGBoost	RF	AdaBoost	LightGBM	XGBoost	RF	AdaBoost	LightGBM
R^2^	0.950	0.928	0.708	0.818	0.842	0.823	0.762	0.825
RMSE	2.155	2.591	5.217	4.121	4.285	4.538	5.26	4.515
MAE	1.569	2.009	4.223	3.163	3.182	3.581	4.314	3.441
a10-index	0.926	0.822	0.509	0.718	0.659	0.585	0.439	0.585

## Data Availability

Not applicable.

## Appendix A. All test data

**Author**	**Literature**	**ID**	**Kind**	**Size(μm)**	**W/B**	**MRR(%)**	**fc(Mpa)**
Zhang Xiu qin	EXPERI MENTAL STUDY ON THE UTILIZATION OF RENEWA BLE MICRON (Chinese) [74]	1	RCP	0.00	0.50	0.00	42.70
		2	RCP	29.78	0.50	10.00	39.10
		3	RCP	29.78	0.50	20.00	35.80
		4	RCP	29.78	0.50	30.00	31.20
Shujun Li et al.	Experimental Study on the Preparation of Recycled Admixtures by Using Construction and Demolition Waste [39]	5	RCP	0.00	0.50	0.00	46.60
		6	RCP	22.50	0.50	30.00	33.55
		7	RCP	37.50	0.50	30.00	32.62
		8	RBP	22.50	0.50	30.00	36.35
		9	RBP	37.50	0.50	30.00	34.48
Lan Cong et al.	Study on the Application of Recycled Fine Powder in Ready-Mixed Concrete [40]	10	RBP	16.61	0.50	30.00	33.05
		11	RBP	14.44	0.50	30.00	34.99
		12	RBP	12.67	0.50	30.00	37.42
		13	RBP	10.66	0.50	30.00	37.91
Wang Hua	INFLUENCE OF RECYCLED FINE POWDER ON SHRINKAGE CRACKING OF CONCRETE (Chinese) [75]	14	RCP	100.00	0.50	30.00	29.90
		15	RCP	70.00	0.50	30.00	32.30
		16	RCP	50.00	0.50	30.00	30.20
Liu Rongtao	Experimental Study on the Construction Waste Clay Brick Powder as Active Admixture (Chinese) [76]	17	RBP	25.00	0.50	30.00	28.70
		18	RBP	15.00	0.50	30.00	31.90
		19	RBP	10.00	0.50	30.00	32.70
		20	RBP	0.00	0.50	0.00	42.50
		21	RBP	15.00	0.50	10.00	38.30
		22	RBP	15.00	0.50	20.00	35.70
		23	RBP	15.00	0.50	30.00	31.90
		24	RBP	15.00	0.50	40.00	26.30
		25	RBP	15.00	0.50	50.00	22.10
		26	RBP	15.00	0.50	60.00	16.60
Kang Xiaoming	Study on the Influence of the Particle Size Distribution of Recycled Concrete Powder on the Mechanical Properties and Microstructure of Rcycled Mortar (Chinese) [77]	27	RCP	34.82	0.50	10.00	42.00
		28	RCP	34.82	0.50	20.00	40.30
		29	RCP	34.82	0.50	30.00	31.00
		30	RCP	19.40	0.50	10.00	43.50
		31	RCP	19.40	0.50	20.00	41.00
		32	RCP	19.40	0.50	30.00	31.50
		33	RCP	18.53	0.50	10.00	50.60
		34	RCP	18.53	0.50	20.00	44.00
		35	RCP	18.53	0.50	30.00	36.10
		36	RCP	0.00	0.50	0.00	50.80
		37	RCP	67.79	0.50	10.00	45.50
		38	RCP	67.79	0.50	20.00	37.20
		39	RCP	67.79	0.50	30.00	26.80
		40	RCP	67.79	0.50	40.00	17.60
Xu Changwei et al.	Study on activation of waste clay brick powder [41]	41	RBP	14.71	0.50	30.00	23.42
		42	RBP	13.89	0.50	30.00	25.30
		43	RBP	12.85	0.50	30.00	27.42
		44	RBP	0.00	0.50	0.00	40.90
		45	RBP	12.85	0.50	20.00	48.10
		46	RBP	12.85	0.50	30.00	39.20
		47	RBP	12.85	0.50	40.00	31.80
		48	RBP	12.85	0.50	50.00	26.50
		49	RBP	12.85	0.50	60.00	21.10
XU Changwei et al.	Application of waste clay brick powder in grouting material (Chinese) [78]	50	RBP	20.90	0.50	30.00	22.10
		51	RBP	16.70	0.50	30.00	27.70
		52	RBP	14.37	0.50	30.00	32.90
		53	RBP	12.71	0.50	30.00	35.60
Zheng Li	Properties of Concrete with Recycled Clay- Brick-Powder (Chinese) [79]	54	RBP	100.00	0.50	30.00	44.90
		55	RBP	60.00	0.50	30.00	45.10
		56	RBP	40.00	0.50	30.00	45.10
		57	RBP	100.00	0.50	20.00	45.04
		58	RBP	60.00	0.50	20.00	48.45
		59	RBP	40.00	0.50	20.00	49.10
		60	RBP	100.00	0.50	10.00	48.90
		61	RBP	60.00	0.50	10.00	52.00
		62	RBP	40.00	0.50	10.00	50.10
WANG Yuan-yuan et al.	STUDY ON MECHANICAL PROPERTIES OF WASTE CLAY-BRICK-POWDER MORTAR (Chinese) [80]	63	RBP	0.00	0.35	0.00	54.90
		64	RBP	53.00	0.35	10.00	50.00
		65	RBP	53.00	0.35	20.00	48.30
		66	RBP	53.00	0.35	30.00	45.10
		67	RBP	71.00	0.35	10.00	52.30
		68	RBP	71.00	0.35	20.00	48.20
		69	RBP	71.00	0.35	30.00	45.10
		70	RBP	82.00	0.35	10.00	48.80
		71	RBP	82.00	0.35	20.00	44.90
		72	RBP	82.00	0.35	30.00	44.80
		73	RBP	45.00	0.35	10.00	53.60
		74	RBP	45.00	0.35	20.00	40.00
		75	RBP	45.00	0.35	30.00	37.10
Zhang Ping et al.	Study on the method of stimulating the activity of regenerated micro powder (Chinese) [81]	76	RCP	0.00	0.50	0.00	49.20
		77	RCP	8.06	0.50	10.00	46.10
		78	RCP	8.06	0.50	20.00	44.80
		79	RCP	8.06	0.50	30.00	43.10
		80	RCP	8.06	0.50	40.00	33.40
		81	RCP	8.06	0.50	50.00	28.00
Liu Yin et al.	Experimental Research on Cementitious Property of Renewable Powders of Construction Waste (Chinese) [82]	82	RCP	0.00	0.50	0.00	30.60
		83	RCP	50.00	0.50	10.00	29.50
		84	RCP	50.00	0.50	20.00	26.90
		85	RCP	50.00	0.50	30.00	23.50
		86	RCP	50.00	0.50	40.00	20.10
Ma Yu	Experimental study on properties of recycled micro powder concrete mixed with construction waste (Chinese) [83]	87	RCP	0.00	0.50	0.00	56.80
		88	RCP	35.00	0.50	10.00	53.10
		89	RCP	35.00	0.50	20.00	50.80
		90	RCP	35.00	0.50	30.00	39.20
		91	RCP	35.00	0.50	40.00	33.60
		92	RCP	35.00	0.50	50.00	25.80
Fan Yao-hu et al.	Effect of Regenerated Powder and Fly Ash on Mechanical Properties and Microstructure of Mortar (Chinese) [84]	93	RCP	0.00	0.50	0.00	49.30
		94	RCP	12.86	0.50	10.00	43.60
		95	RCP	12.86	0.50	20.00	42.50
		96	RCP	12.86	0.50	30.00	33.60
		97	RCP	12.86	0.50	40.00	27.80
Gao shaobin	Full-component of Waste Cement and Utilization of Recycled Concrete (Chinese) [85]	98	RCP	0.00	0.55	0.00	52.50
		99	RCP	24.01	0.55	10.00	47.50
		100	RCP	24.01	0.55	20.00	44.90
		101	RCP	24.01	0.55	30.00	38.20
Zhenhua Duan et al.	Combined use of recycled powder and recycled coarse aggregate derived from construction and demolition waste in self-compacting concrete [37]	102	RCP	0.00	0.50	0.00	42.53
		103	RCP	45.00	0.50	30.00	34.02
		104	RCP	45.00	0.40	0.00	48.41
		105	RCP	45.00	0.40	10.00	42.41
		106	RCP	45.00	0.40	20.00	41.25
Dae-Joong Moon et al.	Fundamental properties of mortar containing waste concrete powder [38]	107	RCP	19.67	0.55	0.00	54.10
		108	RCP	44.12	0.55	20.00	41.90
		109	RCP	20.76	0.55	10.00	52.60
		110	RCP	20.76	0.55	20.00	46.70
		111	RCP	20.76	0.55	30.00	36.40
		112	RCP	18.93	0.55	10.00	50.10
		113	RCP	18.93	0.55	20.00	43.40
		114	RCP	18.93	0.55	30.00	37.30
Shujun Li et al.	Particle-size effect of recycled clay brick powder on the pore structure of blended cement paste [86]	115	RBP	0.00	0.50	30.00	48.30
		116	RBP	25.00	0.50	30.00	39.80
		117	RBP	45.00	0.50	30.00	36.20
		118	RBP	75.00	0.50	30.00	33.70
Zhiming Ma et al.	Mechanical properties and water absorption of cement composites with various fineness and contents of waste brick powder from C&D waste [87]	119	RBP	0.00	0.50	0.00	42.10
		120	RBP	6.00	0.50	7.50	43.30
		121	RBP	6.00	0.50	15.00	44.50
		122	RBP	6.00	0.50	30.00	38.00
		123	RBP	12.00	0.50	30.00	42.60
		124	RBP	12.00	0.50	30.00	43.40
		125	RBP	12.00	0.50	30.00	37.80
		126	RBP	18.00	0.50	30.00	40.70
		127	RBP	18.00	0.50	30.00	39.10
		128	RBP	18.00	0.50	30.00	36.30
		129	RBP	42.00	0.50	30.00	38.50
		130	RBP	42.00	0.50	30.00	37.40
		131	RBP	42.00	0.50	30.00	32.90
Huixia Wu et al.	Water transport and resistance improvement for the cementitious composites with eco-friendly powder from various concrete wastes [88]	132	RCP	0.00	0.50	0.00	42.50
		133	RCP	9.00	0.50	10.00	37.90
		134	RCP	9.00	0.50	20.00	35.40
		135	RCP	9.00	0.50	30.00	32.13
		136	RCP	9.00	0.50	50.00	20.70
Huixia Wu et al.	Properties of green mortar blended with waste concrete-brick powder at various components, replacement ratios and particle sizes [89]	137	RCP	0.00	0.50	0.00	42.50
		138	RCP	14.30	0.50	10.00	37.60
		139	RCP	14.30	0.50	20.00	34.30
		140	RCP	14.30	0.50	30.00	30.50
		141	RBP	11.80	0.50	10.00	41.30
		142	RBP	11.80	0.50	20.00	38.70
		143	RBP	11.80	0.50	30.00	34.90
Zhenhua Duan et al.	Study on the essential properties of recycled powders from construction and demolition waste [90]	144	RBP	0.00	0.50	0.00	45.00
		145	RBP	12.64	0.50	10.00	41.40
		146	RBP	12.64	0.50	20.00	40.70
		147	RBP	12.64	0.50	30.00	37.80
Shujun Li et al.	Investigation of using recycled powder from the preparation of recycled aggregate as a supplementary cementitious material [91]	148	RCP	0.00	0.50	0.00	47.10
		149	RCP	9.00	0.50	30.00	35.32
		150	RCP	14.00	0.50	30.00	34.40
		151	RCP	18.00	0.50	30.00	32.10
		152	RCP	28.00	0.50	30.00	31.60
		153	RBP	9.30	0.50	30.00	37.20
		154	RBP	13.00	0.50	30.00	36.50
		155	RBP	20.00	0.50	30.00	34.10
		156	RBP	27.00	0.50	30.00	33.90
Xiao-xiao Yu	Effect of Mechanical Force Grinding on the Properties of Recycled Powder (Chinese) [92]	157	RCP	27.50	0.50	5.00	42.40
		158	RCP	27.50	0.50	10.00	41.50
		159	RCP	27.50	0.50	15.00	37.80
		160	RCP	27.50	0.50	20.00	34.80
		161	RCP	27.50	0.50	25.00	32.40
		162	RCP	27.50	0.50	30.00	30.30
		163	RCP	27.50	0.50	35.00	23.80
		164	RCP	27.50	0.50	40.00	20.00
		165	RCP	27.50	0.50	45.00	16.30
		166	RCP	27.50	0.50	50.00	12.50
		167	RCP	27.50	0.50	55.00	11.20
		168	RCP	27.50	0.50	60.00	5.40
		169	RCP	32.50	0.50	5.00	41.60
		170	RCP	32.50	0.50	10.00	38.20
		171	RCP	32.50	0.50	15.00	36.10
		172	RCP	32.50	0.50	20.00	32.50
		173	RCP	32.50	0.50	25.00	30.10
		174	RCP	32.50	0.50	30.00	36.90
		175	RCP	32.50	0.50	35.00	22.50
		176	RCP	32.50	0.50	40.00	17.40
		177	RCP	32.50	0.50	45.00	12.40
		178	RCP	32.50	0.50	50.00	11.30
		179	RCP	32.50	0.50	55.00	11.10
		180	RCP	32.50	0.50	60.00	2.50
Li Zhong	Effect of recycled fine powder/aggregate modification on cement-basedmaterials and its application (Chinese) [93]	181	RCP	0.00	0.50	0.00	43.90
		182	RCP	22.70	0.50	10.00	43.50
		183	RCP	22.70	0.50	30.00	33.30
		184	RCP	22.70	0.50	50.00	20.50
Yang Lin	INVESTIGATION ON R ECYCLED CEMENTITIOUS MATERIALS PR EPAR ING WITH RECYCLED CONCR ETE POWDER (Chinese) [94]	185	RCP	0.00	0.50	0.00	45.20
		186	RCP	33.20	0.50	30.00	27.80
		187	RCP	27.60	0.50	30.00	28.90
		188	RCP	20.60	0.50	30.00	31.30
		189	RCP	16.50	0.50	30.00	29.60
		190	RCP	14.30	0.50	30.00	28.00
		191	RCP	10.30	0.50	30.00	25.70
Huixia Wu et al.	Early-age behavior and mechanical properties of cement-based materials with various types and fineness of recycled powder [95]	192	RCP	0.00	0.50	0.00	38.80
		193	RCP	12.40	0.50	7.00	38.50
		194	RCP	12.40	0.50	15.00	36.40
		195	RCP	12.40	0.50	30.00	30.20
		196	RCP	12.40	0.50	40.00	24.10
		197	RCP	23.50	0.50	7.00	27.80
		198	RCP	23.50	0.50	15.00	34.50
		199	RCP	23.50	0.50	30.00	29.60
		200	RCP	23.50	0.50	40.00	22.90
		201	RCP	103.60	0.50	7.00	35.20
		202	RCP	103.60	0.50	15.00	30.20
		203	RCP	103.60	0.50	30.00	25.20
		204	RCP	103.60	0.50	40.00	20.00

## Appendix B. ML training set data

**ID**	**Kind**	**Size (μm)**	**W/B**	**MRR (%)**	**fc (Mpa)**
1	0	32.5	0.5	40	17.4
2	0	34.823	0.5	10	42
3	1	45	0.35	10	53.6
4	0	27.5	0.5	55	11.2
5	1	27	0.5	30	33.9
6	0	0	0.5	0	46.6
7	0	37.5	0.5	30	32.62
8	1	71	0.35	10	52.3
9	1	100	0.5	30	44.9
10	0	24.01	0.55	10	47.5
11	1	12.85	0.5	40	31.8
12	0	24.01	0.55	30	38.2
13	0	103.6	0.5	15	30.2
14	1	12	0.5	30	43.4
15	0	45	0.4	20	41.25
16	1	15	0.5	30	31.9
17	1	40	0.5	20	49.1
18	1	15	0.5	10	38.3
19	0	10.3	0.5	30	25.7
20	0	22.5	0.5	30	33.55
21	0	35	0.5	20	50.8
22	1	11.8	0.5	30	34.9
23	1	12.85	0.5	60	21.1
24	0	45	0.4	10	42.41
25	0	22.7	0.5	10	43.5
26	1	75	0.5	30	33.7
27	0	29.78	0.5	20	35.8
28	0	9	0.5	30	35.32
29	0	0	0.5	0	50.8
30	1	12.85	0.5	20	48.1
31	1	18	0.5	30	39.1
32	1	14.44	0.5	30	34.99
33	1	45	0.35	30	37.1
34	1	15	0.5	50	22.1
35	1	0	0.5	0	42.5
36	0	12.4	0.5	30	30.2
37	0	33.2	0.5	30	27.8
38	1	100	0.5	10	48.9
39	0	8.06	0.5	40	33.4
40	0	103.6	0.5	40	20
41	0	22.7	0.5	30	33.3
42	0	0	0.5	0	38.8
43	1	0	0.5	0	45
44	0	50	0.5	20	26.9
45	0	20.76	0.55	10	52.6
46	1	16.7	0.5	30	27.7
47	1	12	0.5	30	42.6
48	0	0	0.5	0	30.6
49	0	35	0.5	40	33.6
50	1	6	0.5	15	44.5
51	0	18.93	0.55	20	43.4
52	1	0	0.5	0	42.1
53	0	32.5	0.5	30	36.9
54	0	14	0.5	30	34.4
55	0	19.403	0.5	30	31.5
56	1	9.3	0.5	30	37.2
57	1	13	0.5	30	36.5
58	1	6	0.5	7.5	43.3
59	1	12	0.5	30	37.8
60	0	0	0.5	0	56.8
61	1	12.85	0.5	30	39.2
62	1	11.8	0.5	10	41.3
63	1	82	0.35	20	44.9
64	0	18.93	0.55	10	50.1
65	1	53	0.35	30	45.1
66	1	25	0.5	30	39.8
67	0	100	0.5	30	29.9
68	0	27.5	0.5	45	16.3
69	0	27.5	0.5	35	23.8
70	1	18	0.5	30	40.7
71	1	42	0.5	30	38.5
72	0	27.6	0.5	30	28.9
73	0	44.12	0.55	20	41.9
74	0	18	0.5	30	32.1
75	0	50	0.5	30	30.2
76	0	35	0.5	10	53.1
77	0	27.5	0.5	50	12.5
78	1	12.64	0.5	20	40.7
79	1	14.71	0.5	30	23.42
80	1	10.66	0.5	30	37.91
81	0	50	0.5	10	29.5
82	0	12.862	0.5	10	43.6
83	1	53	0.35	20	48.3
84	1	6	0.5	30	38
85	0	67.785	0.5	30	26.8
86	0	32.5	0.5	15	36.1
87	0	103.6	0.5	7	35.2
88	0	20.76	0.55	20	46.7
89	1	40	0.5	30	45.1
90	0	67.785	0.5	20	37.2
91	0	32.5	0.5	25	30.1
92	0	20.6	0.5	30	31.3
93	1	45	0.35	20	40
94	0	29.78	0.5	30	31.2
95	0	32.5	0.5	60	2.5
96	0	67.785	0.5	10	45.5
97	0	8.06	0.5	50	28
98	0	32.5	0.5	20	32.5
99	0	12.4	0.5	15	36.4
100	0	9	0.5	50	20.7
101	1	60	0.5	20	48.45
102	1	15	0.5	30	31.9
103	1	12.85	0.5	50	26.5
104	0	32.5	0.5	10	38.2
105	0	12.862	0.5	40	27.8
106	0	35	0.5	50	25.8
107	1	14.37	0.5	30	32.9
108	1	20.9	0.5	30	22.1
109	0	0	0.5	0	45.2
110	0	14.3	0.5	20	34.3
111	0	32.5	0.5	45	12.4
112	1	40	0.5	10	50.1
113	1	82	0.35	10	48.8
114	0	32.5	0.5	35	22.5
115	1	82	0.35	30	44.8
116	0	28	0.5	30	31.6
117	0	0	0.5	0	49.3
118	0	34.823	0.5	30	31
119	0	32.5	0.5	50	11.3
120	0	27.5	0.5	25	32.4
121	1	15	0.5	60	16.6
122	0	18.529	0.5	10	50.6
123	0	27.5	0.5	30	30.3
124	0	8.06	0.5	20	44.8
125	0	0	0.5	0	42.53
126	0	9	0.5	30	32.13
127	0	18.529	0.5	30	36.1
128	0	27.5	0.5	10	41.5
129	0	34.823	0.5	20	40.3
130	0	103.6	0.5	30	25.2
131	0	18.93	0.55	30	37.3
132	0	23.5	0.5	30	29.6
133	0	19.67	0.55	0	54.1
134	0	14.3	0.5	30	30.5
135	0	23.5	0.5	15	34.5
136	0	50	0.5	30	23.5
137	0	45	0.4	0	48.41
138	1	25	0.5	30	28.7
139	0	35	0.5	30	39.2
140	0	0	0.5	0	42.5
141	1	13.89	0.5	30	25.3
142	0	19.403	0.5	10	43.5
143	0	27.5	0.5	20	34.8
144	0	27.5	0.5	5	42.4
145	0	22.7	0.5	50	20.5
146	1	42	0.5	30	37.4
147	1	71	0.35	30	45.1
148	0	23.5	0.5	7	27.8
149	1	11.8	0.5	20	38.7
150	0	23.5	0.5	40	22.9
151	1	37.5	0.5	30	34.48
152	1	60	0.5	30	45.1
153	1	18	0.5	30	36.3
154	0	24.01	0.55	20	44.9
155	1	20	0.5	30	34.1
156	0	9	0.5	10	37.9
157	0	0	0.5	0	42.7
158	1	100	0.5	20	45.04
159	0	12.4	0.5	7	38.5
160	0	12.862	0.5	30	33.6
161	1	0	0.5	30	48.3
162	0	70	0.5	30	32.3
163	0	0	0.5	0	49.2

## Appendix C. ML test set data

**ID**	**Kind**	**Size (μm)**	**W/B**	**MRR (%)**	**fc (Mpa)**
1	0	18.529	0.5	20	44
2	0	19.403	0.5	20	41
3	0	0	0.5	0	47.1
4	0	0	0.5	0	43.9
5	0	27.5	0.5	60	5.4
6	0	0	0.55	0	52.5
7	1	45	0.5	30	36.2
8	1	12.67	0.5	30	37.422
9	1	15	0.5	40	26.3
10	1	12.64	0.5	30	37.8
11	1	12.71	0.5	30	35.6
12	0	8.06	0.5	10	46.1
13	1	12.85	0.5	30	27.4176
14	0	67.785	0.5	40	17.6
15	1	12.64	0.5	10	41.4
16	0	27.5	0.5	40	20
17	1	0	0.5	0	40.9
18	0	29.78	0.5	10	39.1
19	1	16.61	0.5	30	33.048
20	0	45	0.5	30	34.02
21	1	0	0.35	0	54.9
22	0	0	0.5	0	42.5
23	1	10	0.5	30	32.7
24	1	42	0.5	30	32.9
25	0	12.4	0.5	40	24.1
26	0	32.5	0.5	5	41.6
27	0	32.5	0.5	55	11.1
28	0	9	0.5	20	35.4
29	0	14.3	0.5	10	37.6
30	0	14.3	0.5	30	28
31	1	71	0.35	20	48.2
32	1	53	0.35	10	50
33	0	8.06	0.5	30	43.1
34	1	15	0.5	20	35.7
35	0	50	0.5	40	20.1
36	0	27.5	0.5	15	37.8
37	1	22.5	0.5	30	36.348
38	1	60	0.5	10	52
39	0	12.862	0.5	20	42.5
40	0	20.76	0.55	30	36.4
41	0	16.5	0.5	30	29.6
